# Incorporation of black phosphorus nanosheets into poly(propylene fumarate) biodegradable bone cement to enhance bioactivity and osteogenesis

**DOI:** 10.1186/s13018-024-04566-6

**Published:** 2024-01-30

**Authors:** Jiahan Chen, Xiaoxia Huang, Jianghua Wang, Wen Chen, Yong Teng, Dongfeng Yin

**Affiliations:** 1 Graduate School of Xinjiang Medical University, Urumqi, Xinjiang China; 2 Department of Orthopedics, General Hospital of Xinjiang Military Region, Urumqi, Xinjiang China; 3 Department of Pharmacy, General Hospital of Xinjiang Military Region, Urumqi, Xinjiang China; 4 Shihezi University College of Pharmacy, Shihezi, Xinjiang China

**Keywords:** Bone cement, Poly(propylene fumarate), Black phosphorus nanomaterials, Bone regeneration

## Abstract

**Background:**

Injectable bone cement is commonly used in clinical orthopaedics to fill bone defects, treat vertebral compression fractures, and fix joint prostheses during joint replacement surgery. Poly(propylene fumarate) (PPF) has been proposed as a biodegradable and injectable alternative to polymethylmethacrylate (PMMA) bone cement. Recently, there has been considerable interest in two-dimensional (2D) black phosphorus nanomaterials (BPNSs) in the biomedical field due to their excellent photothermal and osteogenic properties. In this study, we investigated the biological and physicochemical qualities of BPNSs mixed with PPF bone cement created through thermal cross-linking.

**Methods:**

PPF was prepared through a two-step process, and BPNSs were prepared via a liquid phase stripping method. BP/PPF was subsequently prepared through thermal cross-linking, and its characteristics were thoroughly analysed. The mechanical properties, cytocompatibility, osteogenic performance, degradation performance, photothermal performance, and in vivo toxicity of BP/PPF were evaluated.

**Results:**

BP/PPF exhibited low cytotoxicity levels and mechanical properties similar to that of bone, whereas the inclusion of BPNSs promoted preosteoblast adherence, proliferation, and differentiation on the surface of the bone cement. Furthermore, 200 BP/PPF demonstrated superior cytocompatibility and osteogenic effects, leading to the degradation of PPF bone cement and enabling it to possess photothermal properties. When exposed to an 808-nm laser, the temperature of the bone cement increased to 45–55 °C. Furthermore, haematoxylin and eosin-stained sections from the in vivo toxicity test did not display any anomalous tissue changes.

**Conclusion:**

BP/PPF exhibited mechanical properties similar to that of bone: outstanding photothermal properties, cytocompatibility, and osteoinductivity. BP/PPF serves as an effective degradable bone cement and holds great potential in the field of bone regeneration.

**Supplementary Information:**

The online version contains supplementary material available at 10.1186/s13018-024-04566-6.

## Background

Bones have essential roles in providing a framework for soft tissue attachment and allowing physical movement, as well as protecting internal organs from damage. Furthermore, bones have a robust regenerative function [1]. However, larger bone defects, known as critical size defects (CSDs), cannot regenerate self-tissue and require surgical intervention. Nonetheless, the current surgical clinical treatment has drawbacks [2].

Injectable bone cement is extensively used in orthopaedics to fill bone defects and provide stability to fractures. It is also used in the treatment of vertebral compression fractures in elderly patients and for the fixation of joint prostheses during joint replacement surgery [3–5]. Over the last few decades, polymethylmethacrylate (PMMA) has been predominantly used as a component of bone cement. Despite being biologically inert, it cannot effectively integrate with bone tissue and may lead to prosthesis loosening [6–8]. However, PMMA has several drawbacks, including damaging the cell curing temperature, mechanical property mismatch in bone, and poor bone conductivity [6, 9, 10]. Additionally, inaccurate composition and preparation may cause toxic effects [11]. Numerous modifiers, such as graphene oxide, chitosan, magnesium, calcium phosphate, and various antibiotics, have been used to improve the biological and degradation properties of PMMA bone cement [11–14]; however, most of the modified complexes have defects that hinder their clinical application. Therefore, researchers are currently seeking alternative materials for bone cement. These materials should be biocompatible, degradable, absorbable by the human body and possess specific mechanical properties, such as those found in calcium phosphate, calcium sulphate, and poly(propylene fumarate) (PPF) bone cement [15–17].

In recent years, the biodegradable compound PPF has used in bone tissue, the cardiovascular system, and ophthalmology [17]. Thermal cross-linking PPF with *N*-vinyl-2-pyrrolidone (NVP) has biodegradability and mild curing temperature [18]. Fumaric acid and propylene glycol, the degradation products of PPF, are harmless to the human body and have mild curing temperatures and functionalised chemical double bonds, making them a superior option compared to other biological materials [17, 19]. However, PPF lacks bone conductivity and antimicrobial properties; does not promote adhesion, proliferation, and differentiation of bone tissue; and cannot release phosphate and other components necessary for bone during degradation, resulting in incomplete bone defect repair. Therefore, PPF is frequently combined with other osteogenic materials to enhance its cytocompatibility and mechanical properties.

Low-layer black phosphorus nanosheets (BPNSs) represent a new class of two-dimensional nanomaterials that exhibit exceptional photothermal effects, cytocompatibility, antibacterial activity, and degradability. Consequently, BPNSs have been thoroughly researched to evaluate their potential applications in tumour treatment and bone regeneration [20–22]. Recent studies have demonstrated that black phosphorus promotes cellular adhesion and proliferation and exerts significant osteogenic effects [23–26]. Moreover, BP nanosheets can combine with free calcium ions present in the blood and transform them into non-toxic calcium phosphate, which contributes to the promotion of bone regeneration [27].

**Fig. 1:**
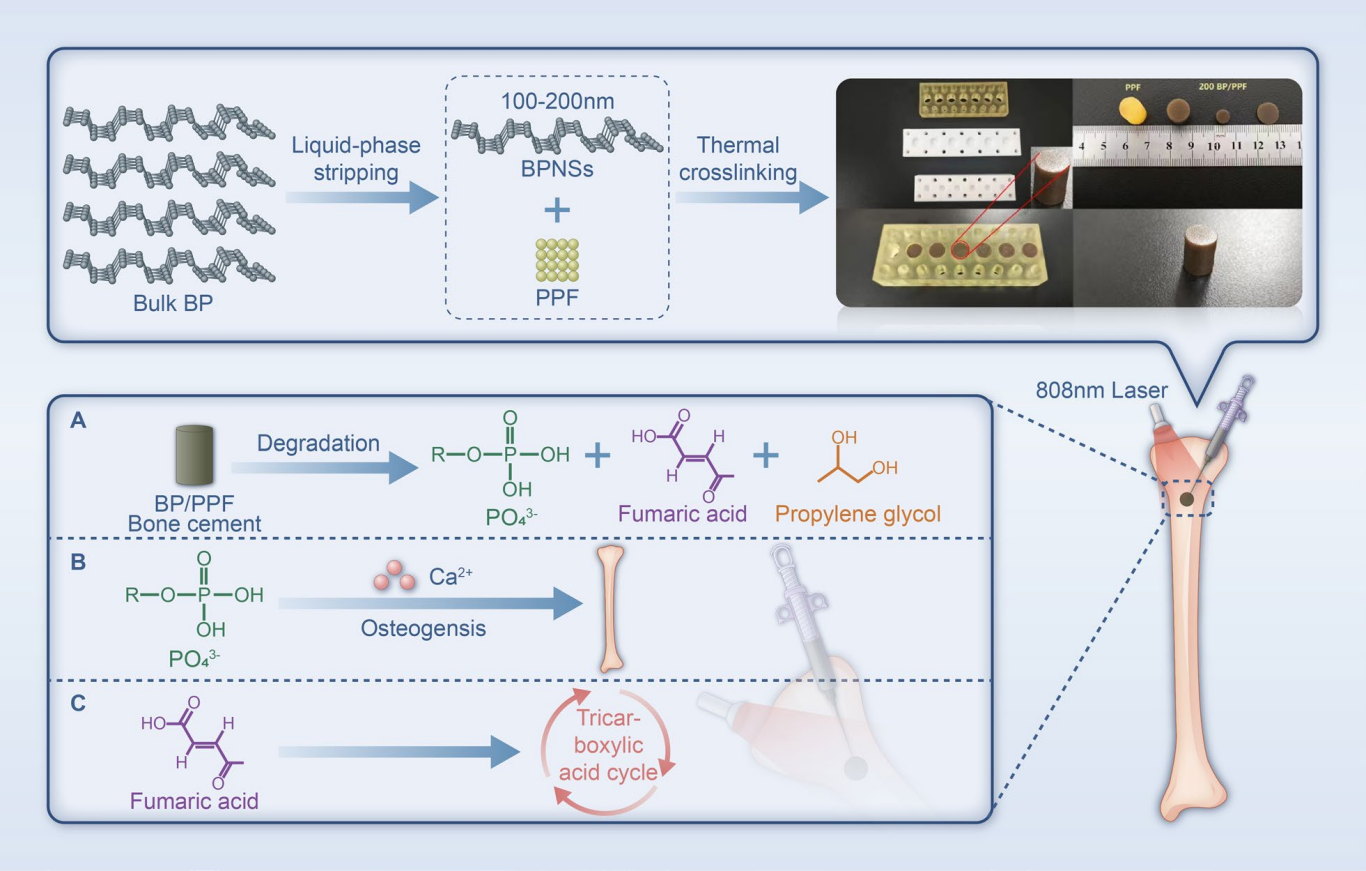
Schematic diagram of the preparation of BP/PPF bone cement

This research involved the generation of BPNSs via the liquid phase stripping process and subsequently incorporating them into the liquid composition of PPF bone cement in a specific proportion followed by thermal cross-linking (Fig. 1). We conducted an analysis of the photothermal properties, phosphate release, mechanical property, in vitro degradability, cytocompatibility, osteogenic induction, and in vivo toxicity of BP/PPF to demonstrate its practicality and feasibility in clinical treatment. Our research aims to highlight the potential applications of BP/PPF and encourage future clinical use.

## Materials and methods

### Preparation of PPF

The synthesis of PPF involved a two-step method [28, 29]. Initially, diethyl fumarate, 1, 2-propylene glycol, hydroquinone, and zinc chloride were mixed in a ratio of 1:3:0.01:0.002 (mol) to establish the reaction system. Subsequently, the system was gradually heated to 150°h and maintained under vacuum for 12 h to produce the PPF reaction product. After cooling, the reaction product was treated with dichloromethane solvent at a 1:1 volume ratio. Impurities were removed, and the product was purified using anhydrous sodium sulphate, hydrochloric acid, and saturated sodium chloride. Then, the purified product was subjected to a rotary evaporator at 40 ry to remove the methylene chloride solvent, resulting in a transparent, light brown and yellow viscous liquid. The gel permeation chromatography (1515 GPC, Waters, USA) (*n* = 3) was used to measure the average number and PPF's molecular weight, as well as its polymerisation degree distribution index. Furthermore, the ^1^HNMR spectrum (Avance III 400, Bruker, GER) was used to determine the structure of the PPF.

### Preparation of BPNSs

BPNSs were produced via a liquid-phase stripping method [30]. In brief, bulk black phosphorus (Macklin, B916424, CN) weighing 40 g was crushed with a quartz bowl and added to 80 mL of N-methylpyrrolidone (NMP) while being shielded by argon in an ice-water bath. The mixture was then subjected to ultrasonic treatment at 250 W for 14 h, followed by simultaneous probe ultrasonic treatment for the same duration at 180 W with a switching period of 3 s/3 s. The mixture was centrifuged at 1073×*g* for 10 min to separate the lower bulk of black phosphorus. Then, it was centrifuged again at 9660×*g* for 20 min to remove the upper layer of NMP. The remaining precipitate was the BPNSs.

### Preparation of BP/PPF

The prepared BPNSs underwent three rounds of rinsing with *N*-vinyl-2-pyrrolidone (NVP) before being redispersed in the NVP in accordance with the proportions listed in Table 1. Next, benzoyl peroxide (BPO) was added to the NVP dispersion and agitated via ultrasound. The resulting mixture was then combined with PPF and stirred magnetically. Finally, the initiator dimethyl 1,4-benzenedicarboxylate (DMT) was added, stirred, and rapidly transferred to a polytetrafluoroethylene (PTFE) mould (*d* = 6 mm), where the sample was cured for 10 min. The formulation designs are detailed in Table 1. The control group comprised PPF bone cement without BPNSs, whereas the other cement containing BPNSs was identified as BP/PPF.

**Table 1:** Composition of BP/PPF Cement

Group	PPF (g)	NVP (ml)	BPO (mg)	DMT (µl)	BP (µg)
PPF	1	0.25	5	1	0
100 BP/PPF	1	0.25	5	1	100
200 BP/PPF	1	0.25	5	1	200
300 BP/PPF	1	0.25	5	1	300

### Characterisation

The molecular structure of PPF was verified through ^1^HNMR analysis. Gel permeation chromatography (*n* = 3) was used to measure the number-average molecular weight, weight-average molecular weight, and degree distribution index of polymerisation of PPF. Furthermore, the morphologies of PPF and BP/PPF were examined using field-emission scanning electron microscopy (SEM, Zeiss Gemini 300, GER). The particle size distributions of the BPNSs were measured using dynamic light scattering (DLS, Zetasizer Ultra, USA). Transmission electron microscopy (TEM, FEI Talos F200X, USA) was used to establish the morphologies of the BPNSs. The crystal structures of the BPNSs were identified and analysed by the X-ray diffractometer (XRD, Rigaku Ultima IV, JP) at a scanning rate of 0.02°/s in two ranges between 10° and 85°. Moreover, the atomic vibration mode of the BPNSs was analysed using Raman spectroscopy (LabAM-HR, FRA). The surface components and their chemical states of the BPNSs were analysed via X-ray photoelectron spectroscopy (XPS, ESCALAB 250Xi, USA). The composition of the BPNSs was examined through Fourier-transform infrared spectroscopy (FT-IR, Thermo Scientific Nicolet iS20, USA).

### Mechanical properties

The specimens (10 mm diameter and 15 mm height) were designed for the compression experiments according to the ISO 604 standard. An electromechanical universal testing machine with a load cell of 30 kN (E44.304, MTS systems (China) LTD, CN) was used to conduct the compression experiment. The compression speed was set to the standard 2 mm/min. Before the compression experiment, the top and bottom surfaces of the specimens were carefully polished with sandpaper to ensure that the upper surfaces were parallel to the lower surfaces.

### Setting properties

The final curing time was recorded using a Vicat apparatus (WKY-1000, Tianjian Instrument Co., LTD, CN), which was calculated as the duration from the cross-linking initiation to the formation of a solidified structure.

### Cell culture

MC3T3-E1 cells were cultured and resuscitated using α-MEM medium containing 10% foetal bovine serum (FBS) and 1% penicillin/streptomycin. For the osteogenic differentiation test, the α-MEM medium was substituted with osteogenic induction medium OriCell, comprising 10% FBS and 1% penicillin/streptomycin, as well as 100 nM dexamethasone, 50 μg/mL AA, and 10 mM β-GP. Prior to culture with cells, the cement samples were immersed in 75% alcohol and air-dried under ultraviolet light.

### Cell adhesion and cytotoxicity assay

First, disinfected discs of PPF and BP/PPF bone cement (diameter = 6 mm, height = 1 cm) were placed in 24-well plates and soaked in α-MEM medium for 30 min. The log-growth process was then initiated. The suspension of mouse preosteoblast cells (MC3T3-E1) was digested using pancreatic enzymes, and 5 × 10^4^ cells per well were uniformly inoculated onto the 24-well plate. Following an incubation period of 24 h, the bone cement was removed and washed with PBS. Fixed in 2.5% glutaraldehyde for 30 min, the samples were subsequently dehydrated using gradients of ethanol (50%, 60%, 70%, 80%, 90%, and 100%). The bone cement followed the same procedure. The cytotoxicity of PPF and BP/PPF regarding mouse preosteoblast cells (MC3T3-E1) underwent an assessment using the CCK-8 assay. The assay involved the placement of disinfected PPF and BP/PPF bone cement discs in 24-well plates for co-culturing with MC3T3-E1 cells (3 × 10^4^ cells/well, *n* = 3). At 1, 3, 5, and 7 days, the CCK-8 detection solution was added, and the absorbance rate at 450 nm was measured via an enzyme labeller (Multiskan SkyHigh, Thermo Fisher Scientific, USA). Accordingly, the cell survival rate was recorded. The experimental group received bone cement, whereas the control group did not. Furthermore, a cell viability/death assay kit was used to stain cells cultured on bone cement PPF and BP/PPF.

The activity state of adherent cells on the surface of the bone cement was observed directly using live/dead cell viability staining. The disinfected PPF and BP/PPF bone cement discs were arranged on a 24-well dish and cultured with MC3T3-E1 cells (3 × 10^4^ cells/well). The bone cement was removed 1, 3, and 5 days after seeding, followed by staining using a LIVE/DEAD cell imaging kit. The resulting images were captured using a fluorescent inverted microscope (Carl Zeiss, Germany).

### Osteogenic differentiation

The process of mineralisation in osteoblasts was evaluated using Alizarin Red staining. Co-culturing with MC3T3-E1 cells (3 × 10^4^ cells/well), the cells were incubated in a 24-well plate equipped with osteogenic induction differentiation medium (OriCell, CN) for 14 days. Next, the culture medium was discarded, and each well was exposed to 2 mL of a 4% paraformaldehyde solution. The specimens were then fixed for 25 min at room temperature and subsequently stained with 2 mL of Alizarin Red at room temperature for 5 min. Calcified nodules were observed and photographed with an optical microscope.

The potential of PPF and BP/PPF to promote osteogenic differentiation over 14 and 21 days was analysed using a Mouse Alkaline Phosphatase (ALP) ELISA Kit (Elabscience, CN, USA) and a Mouse OC/BGP (Osteocalcin) ELISA Kit (Elabscience CN). A standard curve was created as per the kit's instructions, and the absorbance was measured at a 450 nm wavelength using an enzyme labeller. The ALP activity of PPF and BP/PPF was measured on day 14, and the osteocalcin content was determined on day 21 using a standard curve.

### Degradation behaviour

After recording the initial weight (M_0_), the PPF and BP/PPF discs (diameter = 6 mm, height = 1 cm) were submerged in a PBS solution (pH = 7.3, 0.1 g/10 mL) and placed on a shaking table at 37 °C for 3, 7, 14, 21, and 28 days. At regular intervals, the samples were extracted from the solution, washed with deionised water, and dried in a vacuum drying oven. The weight of the sample (M_2_) was then compared to its initial weight (M_0_) to determine the rate of weight loss of the bone cement.

### In vitro photothermal-conversion efficiency

The PPF and BP/PPF discs (diameter = 6 mm, height = 1 cm), which had been prepared in advance, were submerged in 0.5 mL of PBS. Following this, the samples underwent irradiation with an 808 nm laser that had the same power density (1.0 W/cm^2^). The heating curves of the samples were documented using a temperature recorder. To evaluate the stability of the photothermal effect, the 200 BP/PPF bone cement was then irradiated five times (*n* = 3).

### Phosphate release

To ascertain the kinetics of phosphate release, 200 BP/PPF bone cement was divided into two groups (*n* = 3) and placed in 2 mL deionised water glass vials. The vials were then subjected to incubation in a shaker under 37 °C. One group underwent irradiation using an 808 nm laser for 10 min each day (power density of 1.0 W/cm^2^), whereas the other group was not exposed to irradiation. Then, 0.5 mL of solution was withdrawn from the bottle sequentially, and the flask was refilled with fresh deionised water. The extracted solution was assessed for its phosphate concentration on days 1, 3, 5, 7, 10, 14, 21, and 28 using the phosphate assay kit (ab65622, Abcam, Cambridge, UK).

### In vivo toxicity

Forty BALB/c mice (20 females and 20 males) were divided randomly into four groups. Each group of mice (*n* = 10) received intraperitoneal injections of BP/PPF extract liquid every 7 days (50 mL/kg each time), and the animals' overall health was monitored. After 28 days, the mice were sacrificed, and their heart, liver, spleen, lungs, and kidneys were stained with haematoxylin and eosin (H&E) to observe any potential tissue toxicity.

### Statistical analysis

Statistical analyses were performed using SPSS AU (Beijing Green Silk Inc., CN). The data were presented as mean ± standard deviation (*n* = 3). One-way analysis of variance (ANOVA) was used to compare differences between the two groups. In all statistical analyses, statistical significance was established at *p* < 0.05.

## Results

### Characterisation of BP and PPF

**Fig. 2:**
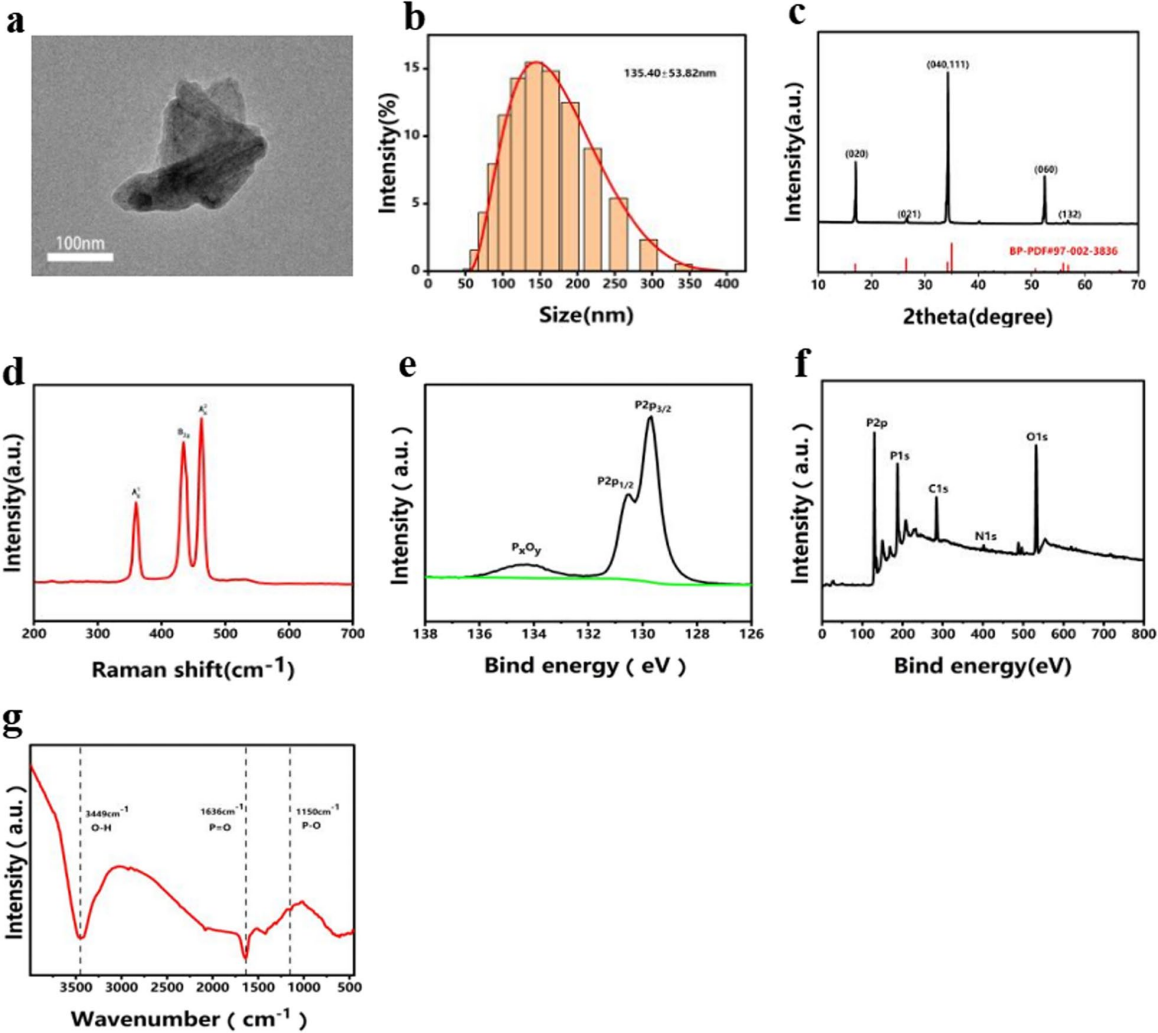
Characterisation of BPNSs: **a** TEM image of BPNSs with a scale bar of 100 nm. **b** DLS size distribution of BPNSs. **c** XRD patterns of BPNSs. **d** Raman scattering spectrum of BPNSs. **e** XPS spectrum and **f** P 2p XPS spectrum of BPNSs. **g** FT-IR spectrum of BPNSs

TEM imaging in Fig. 2a illustrates a thin, lamellar crystal structure with dimensions of 100–200 nm. The DLS outcomes displayed in Fig. 2b indicate that the average transverse size of the BPNSs is 135.40 ± 53.82 nm. In Fig. 2c, the XRD analysis of BPNSs revealed three strong characteristic diffraction peaks at 17.0, 34.2, and 52.3. These peaks corresponded to the crystal faces of black phosphorus (020), (040), and (060), respectively. The absence of characteristic peaks of other impurities indicated the high purity of the BPNSs produced in this study. The XRD analysis was consistent with JCPDS BP-PDF# 97–002-3836. The Raman test results of BPNSs are depicted in Fig. 2d. Three distinct characteristic peaks were observed at 359.9, 434.6, and 462.6 cm^−1^. These peaks corresponded to the three vibration modes of the atomic structures of the BP crystal $A_{g}^{1}$, B2g, and $A_{g}^{2}$, respectively; these results are consistent with those reported in other literature [31]. The XPS spectrum displayed two peaks, at 128.9 eV and 130.1 eV, corresponding to the P2p and P2s peaks of black phosphorus, respectively, affirming that the nanoparticle mainly comprises phosphorus. The segmentation map of P2p, as shown in Fig. 2e, indicated double peaks at 130.1 and 131.2 eV, corresponding to the P2p1/2 and P2P2/3 components, respectively. A phosphorus oxide-related peak emerged at 133.0–134.0 eV. These findings suggest that a minor portion of the BPNS underwent oxidation [32]. The FT-IR spectrum depicted in Fig. 2g substantiates the presence of the O–H peak at 3449 cm^−1^, along with the P=O and P–O peaks appearing at 1636 and 1150 cm^−1^, respectively. The ^1^HNMR spectrum confirmed that the target product was PPF (Additional file 1: Fig. S1, Additional file 2: Fig. S2). Gel filtration chromatography demonstrated that the PPF sample possessed a numerical average molecular weight of 1392 ± 323, a weight-average molecular weight of 2340 ± 403, and a polymerisation distribution index of 1.70 ± 0.18 (Additional file 3: Table S1).

### Characterisation of BP/CPC

**Fig. 3:**
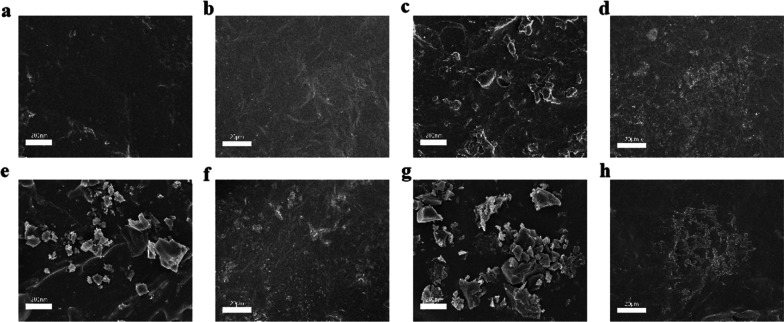
Characterisation of PPF and BP/PPF. Representative SEM images of **a** PPF(Scale bars, 200 nm), **b** PPF(Scale bars, 20 μm), **c** 100 BP/PPF(Scale bars, 200 nm), **d** 100 BP/PPF(Scale bars, 20 μm), **e** 200 BP/PPF(Scale bars, 200 nm), **f** 200 BP/PPF(Scale bars, 20 μm), **g** 200 BP/PPF(Scale bars, 200 nm), **h** 200 BP/PPF(Scale bars, 20 μm)

The PPF and BP/PPF were produced as cylinders measuring 1 cm in height and 6 mm wide before being measured. Figure 3 shows the surface morphology after the preparation. The bone cement that underwent PPF thermal cross-linking displayed a smooth planar structure with no observable flaky particles on the surface. After the addition of BPNSs, the surface of the bone cement transformed into a thin lamellar crystal substance, possibly because of BPNS surface adhesion. At the micron scale, the PPF and BP/PPF surfaces, which were compact and smooth without obvious pore structure, were comparable. The addition of BPNSs did not significantly change the surface roughness of PPF bone cement.

### Mechanical properties

**Fig. 4:**
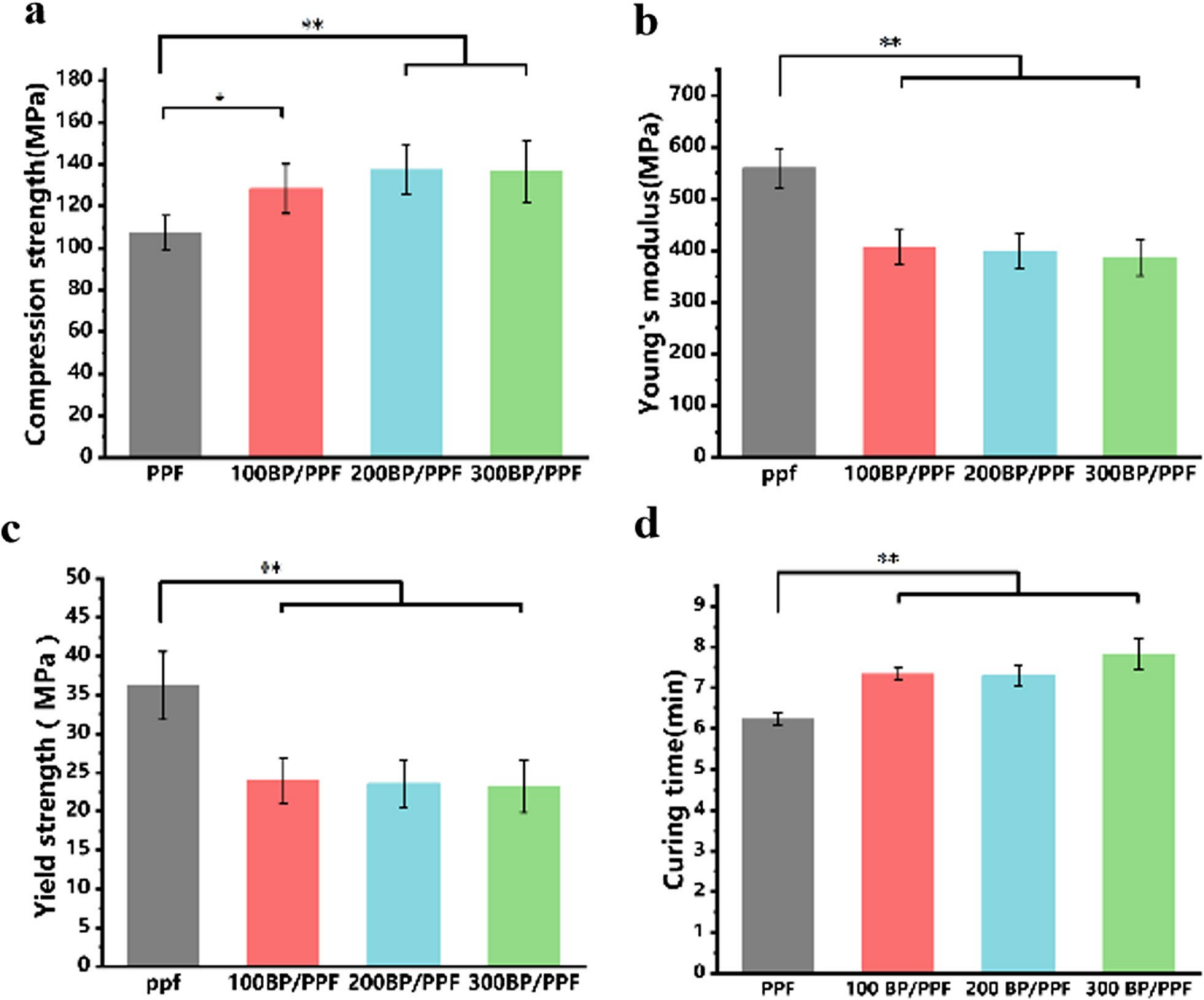
Mechanical properties and curing times of BP/PPF and PPF (*n* = 5, **p < 0.01, *p < 0.05). **a** Compression strength. **b** Young’s Modulus. **c** Yield strength. **d** Curing times

Increasing the BPNS content changes the nature of PPF bone cement (Fig. 4a). The compressive strength of the cement increased from 107.2 ± 8.3 to 128.4 ± 11.8 MPa (*p* < 0.01), whereas the elastic modulus reduced from 559.8 ± 38.1 MPa to 406.6 ± 33.1 MPa (p < 0.01) (Fig. 4b). In addition, the yield strength decreased from 34.2 ± 2.6 MPa to 26.4 ± 2.3 Mpa (*p* < 0.01) (Fig. 4c). There was no significant difference in BP/PPF between the two groups (*p* > 0.05).

### Setting properties

Te curing times of BP/PPF are similar to that of pure PPF (Fig. 4d). As the BPNS content increases, the curing time of bone cement increased slightly from 6.2 min to 7.3 min, but there was no significant difference between the BP/PPF and BP/PPF groups (*p* > 0.05).

### Cell adhesion and cytotoxicity assay

**Fig. 5:**
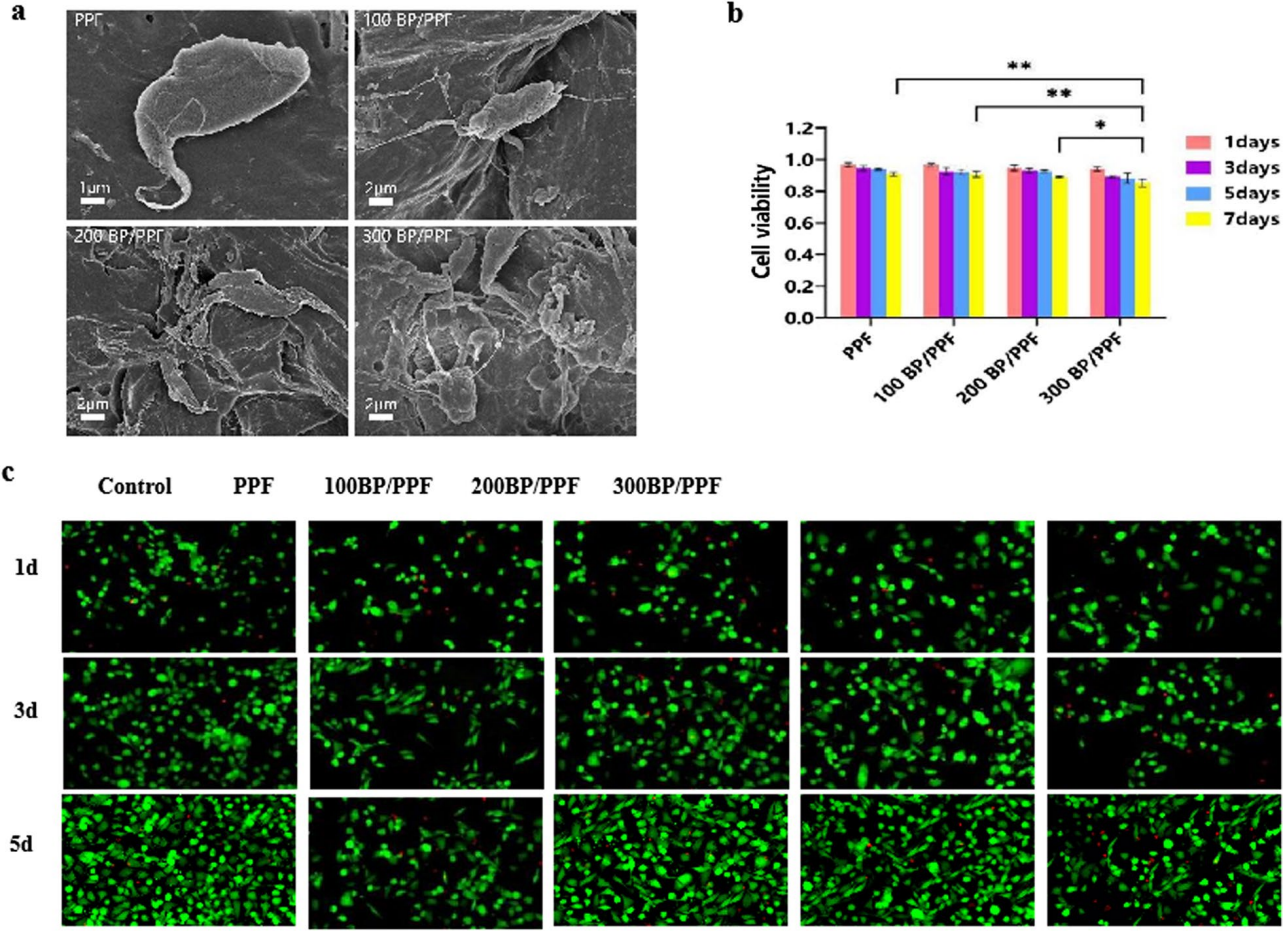
Cytocompatibility assay of PPF and BP/PPF: **a** SEM images show the adhesion of MC3T3-E1 cells to PPF and BP/PPF surfaces after 24 h. **b** Cell viability was assessed through CCK-8 cytotoxicity assays for four types of PPF at 1, 3, 5 and 7 days after seeding (*n* = 3, **p < 0.01, *p < 0.05). **c** Live-dead cell staining of MC3T3-E1 cells on BP/PPF bone cement reveals live cells in green and dead cells in red

Figure 5a, c depicts the adhesion of MC3TE-E1 cells to the bone cement surface. The findings reveal that the number of cell adhesions on the PPF cement surface was remarkably lower than on the BP/PPF cement. As shown in Fig. 5b, the cell survival rate of adherent cells exceeded 80%, thus indicating satisfactory cytocompatibility of bone cement. However, the cell survival rate of bone cement 300 BP/PP was inferior to that of other bone cement, and this difference was statistically significant (*p* < 0.05).

### Osteogenic differentiation

**Fig. 6:**
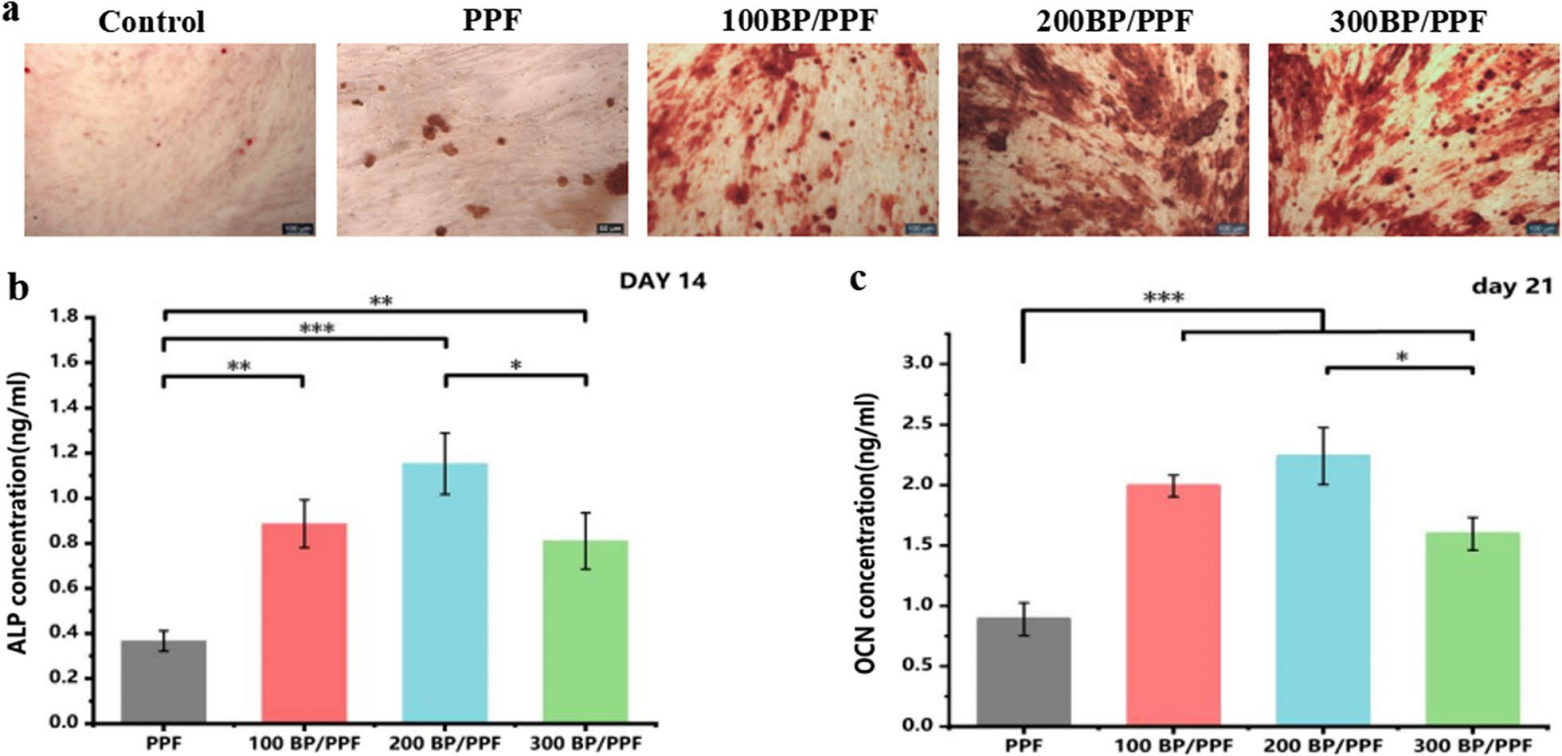
Osteogenic differentiation of PPF and BP/PPF: **a** Alizarin Red S (ARS) was used to stain MC3T3-E1 cells on day 14, with images showing a scale bar of 50 μm. **b** On day 14, ALP activity of MC3T3-E1 cells was measured for four groups of bone cement (*n* = 3, *p < 0.05, **p < 0.01, ***p < 0.001). **c** OCN content of MC3T3-E1 cells from four groups of bone cement on day 21. (*n* = 3, *p < 0.05, **p < 0.01, ***p < 0.001)

As depicted in Fig. 6a, despite the inclusion of osteogenic differentiation induction medium and Alizarin Red staining on day 14, only a few stained calcified nodules were visible in the control and PPF groups. Conversely, a considerably greater amount of stained calcified nodules was detected in the BPNSs group, which was validated by the ALP activity and OCN content. Figure 6b indicates a significant rise in the ALP activity following the addition of BPNSs in contrast to PPF. The ALP activity of the 200 BP/PPF bone cement group was significantly higher than that of the 100 and 300 BP/PPF groups (*p* < 0.05), although no notable differences were observed between the 100 and 300 BP/PPF groups. Furthermore, the application of BPNSs significantly increased the OCN content, with that of the PPF group notably lower (*p* < 0.001) (Fig. 6c). These results indicated that 200 BP/PPF bone cement is the optimal material for promoting osteogenic differentiation in cells.

### Degradation behaviour and photothermal-conversion efficiency of PPF and BP/PPF in Vitro

**Fig. 7:**
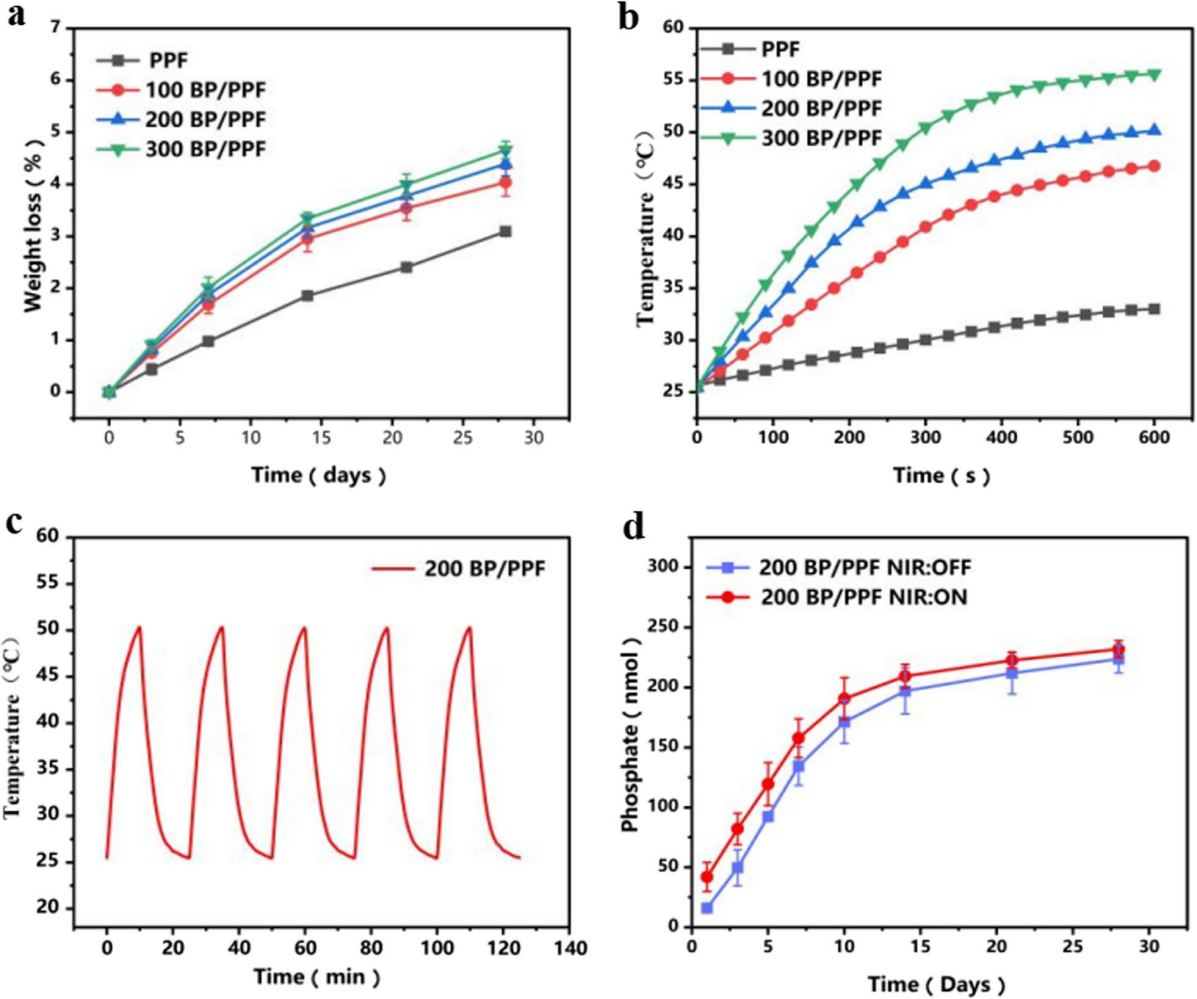
Degradation Behaviour and Photothermal-Conversion efficiency of PPF and BP/PPF in vitro: **a** Evaluation of degradation of BP/PPF composites in PBS in vitro. **b** Examination of photothermal curves of BP/PPF when exposed to 808 nm laser irradiation (power density of 1.0 W/cm^2^). **c** Analysis of photothermal curves of BP/PPF after five NIR cycles (power density of 1.0 W/cm^2^). **d** Assessment of phosphate release kinetics from BP/PPF with and without daily exposure to an 808 nm laser (power density of 1.0 W/cm^2^, laser duration is 10 min per day)

As shown in Fig. 7a, BP/PPF degraded slightly faster than PPF bone cement. Although the degradation rate of BP/PPF was faster during the first two weeks, it then became similar to that of PPF.

When subjected to NIR irradiation at 808 nm and a power density of 1.0 W/cm^2^ (Fig. 7b), the temperature increased from 25 °C to 40–50 °C in 10 min. However, at the same power, the temperature of PPF without BPNS only increased by 5 °C. This result demonstrates the crucial function of BPNSs in photothermal-conversion efficiency. Furthermore, throughout the five photothermal cycles, the photothermal characteristics of BP/PPF remained unaltered (power density of 1.0 W/cm^2^, Fig. 7c).

### Phosphate release capacity of BP/PPF

As depicted in Fig. 7d, the phosphate release in the presence of NIR irradiation was marginally greater than that in the absence of irradiation over the initial two weeks, indicating that NIR irradiation accelerated the conversion of BPNS to phosphate. Significant phosphate release occurred in both experimental groups within two weeks. Subsequently, a decrease in phosphate release was observed after immersion in deionised water for two weeks; however, a small amount of phosphate accumulation was still present.

### In vivo toxicity

**Fig. 8:**
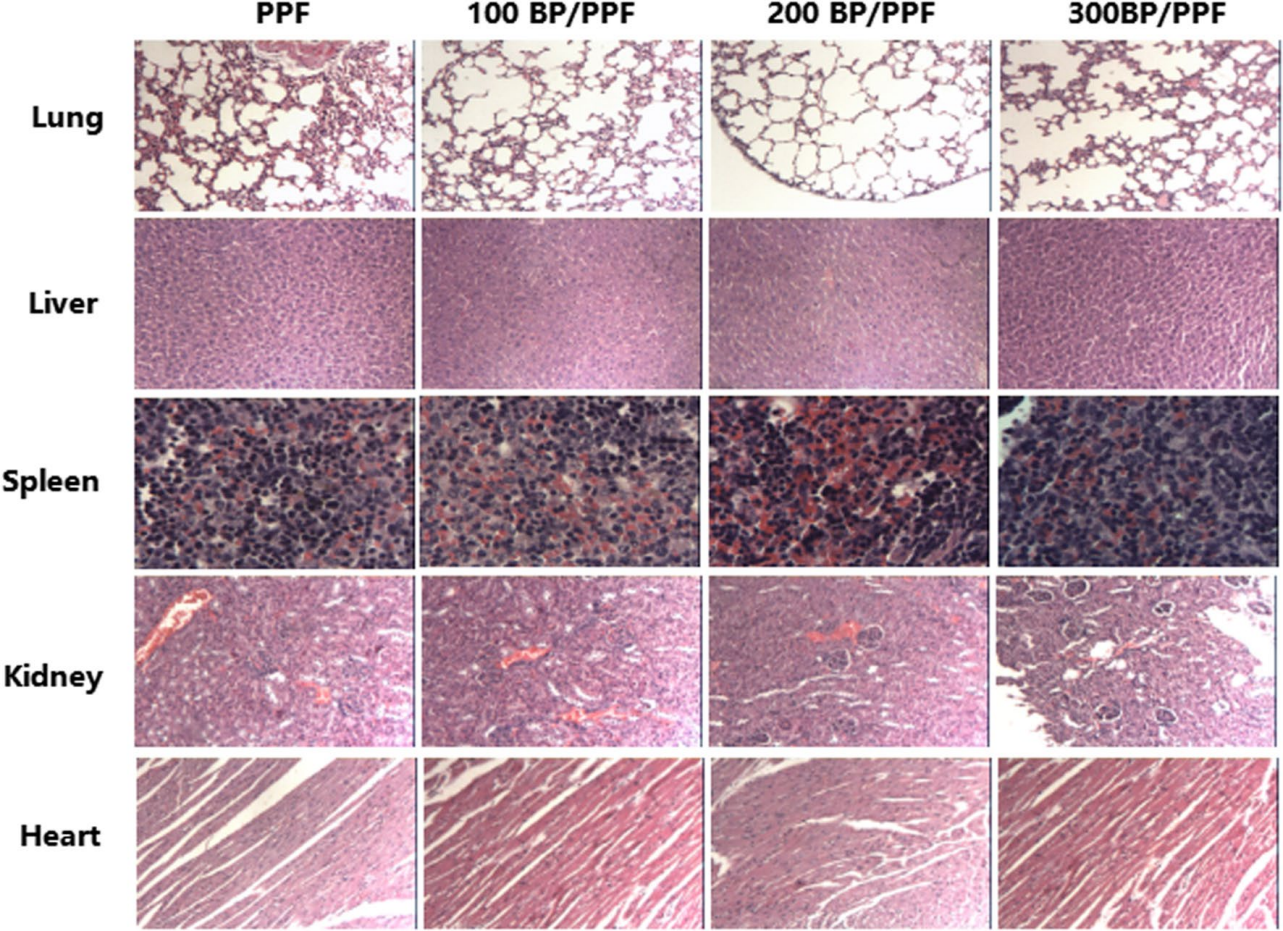
Pathological evaluation, using haematoxylin and eosin-stained images, was conducted on the lungs, liver, spleen, kidney, and heart of mice treated with BPNSs 28 days after injection

In vivo toxicity studies indicated that the mice maintained good health for 28 days following injection with no discernible abnormalities in their diet, sleep patterns, or body temperature. Subsequently, on day 28, the mice were humanely euthanised and subjected to HE staining. The HE staining results (Fig. 8) indicated normal tissue condition in the heart, liver, spleen, lung, and kidney tissues of the three groups of bone cement extract (*n* = 30) and the normal saline control group (*n* = 10). No toxic abnormalities, such as vacuoles, oedema, or necrosis, were noted. The study demonstrated good cytocompatibility of the bone cement BP/PPF in animals, with no evident toxicity or side effects in vivo in accordance with the CCK-8 cytotoxicity assay results.

## Discussion

Regenerative repair of extensive bone defects caused by bone tumours, trauma, and chronic osteomyelitis necessitates the use of bone grafts to replace the diseased or absent bone with either natural or synthetic bone [33–35]. If the filling of bone defects is considered, possible filling options include cement, autologous bone grafts, allogeneic grafts, xenografts, and bone graft substitutes, such as bioceramics, bioglass, and synthetic polymers [36, 37]. In a previous study, we investigated various PPF-HA composite formulations' effects on intervertebral cage mechanical properties, confirming PPF's efficacy as an easily modifiable material with excellent mechanical properties [38]. Throughout extensive exploration, PPF has emerged as an outstanding biodegradable scaffold material for bone tissue engineering and drug delivery [28, 39].

When the average molecular weight of PPF falls between 1000 and 2000, the compressive strength of PPF bone cement ranges between 50 and 120 MPa [40]. The mechanical properties of PPF cement are akin to those of cancellous and cortical bone [41], and PPF displays efficient injectable properties [40, 42]. For patients suffering from osteoporotic vertebral compression fractures, unbiased medical studies have demonstrated that the possibility of adjacent segment disease is greater in patients treated with PMMA bone cement than in those treated with other treatments [10, 43, 44]. The use of PPF as a substitute material for PMMA offers a resolution to the issues caused by the latter's high mechanical strength.

In this study, we found that the addition of BPNS changed the mechanical properties of PPF cement. The bone cement with BPNS exhibited higher compressive strength, which meant that the bone cement was less prone to breakup by excessive external force, and the compressive strength of PPF and BP/PPF cement was close to that of cortical bone (100–180 MPa) [41, 45]. Although the elastic modulus of cement decreases after the addition BPNS, it is still within the range of the elastic modulus of cancellous bone (100–500 MPa) [45]. The similar elastic modulus does not produce stress shielding effect [46, 47], thus avoiding prosthesis loosening and osteoporosis caused by uneven stress distribution after the material is implanted into the human body. The yield strength of BP/PPF cement is slightly lower compared to pure PPF cement, indicating that excessive force is more likely to cause deformation of BP/PPF cement; however, BPNSs exhibit enhanced resistance to disintegration. The change in the BPNS concentration did not significantly affect the mechanical properties of bone cement; these results were consistent with those of previous studies [16]. This may be due to the fact that PPF does not have the same irregular pore structure as PMMA bone cement [11, 48]. PPF bone cement is a dense structure, and its pores are mainly circular bubbles caused by cross-linking heat release and syringe injection. The addition of BPNS does not change the PPF bone cement porosity. Rapid in situ treatment is one of the advantages of injectable bone cement, which can shorten operation time and reduce the pain of patients. Therefore, proper curing time is necessary. We found that the curing time of PPF bone cement was approximately 6 min, and the addition of BPNS slightly increased the curing time of bone cement, which was due to the inorganic material that may inhibit polymerization [49].

The development of PFF has encountered several challenges, including the intricacy of its modification, the challenge of eliminating by-products, reduced mechanical strength after synthesis, and lengthy cross-linking duration. Incorporating nano-sized BPNSs in the study augments the advantages of BP/PPF bone cement while maintaining its original features, as BPNSs minimally affect thermal cross-linking itself. Black phosphorus can easily undergo oxidation to form phosphate compounds when water and oxygen are present [50, 51]. The experimental data reveal that the BP/PPF cement exhibited considerable release of phosphate within two weeks of testing under physiological conditions. This was due to the oxidation of BPNSs immobilised on the cement surface. Although the release rate of phosphate diminished gradually, it continued to accumulate over time. This could be because, during the degradation of PPF, the deeper layers of BPNSs are granted the chance to undergo oxidation, which is in line with prior research suggesting that bone cement is primarily subject to surface corrosion rather than internal degradation and, thus, considered a compact structure [49]. Consequently, the inclusion of BPNSs might generate minor surface pore formations on bone cement, enlarge the cement's outer surface, and expedite the degradation process. Furthermore, the BP/PPF bone cement exhibits excellent light transmission. This property enables BPNSs to effectively use their photothermal performance advantages even in the absence of contact with oxygen and water. The study demonstrated that the BP/PPF cement did not experience photothermal loss due to the excessive degradation of BPNSs following multiple photothermal cycles [16, 52]. This could be due to the fact that most BPNSs are encased in PPF bone cement, which does not oxidise upon contact with oxygen and water.

The cytocompatibility of PPF and BP/PPF plays a crucial role in promoting bone and tissue regeneration. Prior research has demonstrated that BPNSs possess a nanoknife effect and generate reactive oxygen species (ROS) that can inflict cellular damage [53–55]. The material's toxicity was the basis for further research, and our team demonstrated that the BP/PPF bone cement is not very cytotoxic at 200 ppm. SEM and live/dead cell staining revealed that MC3T3-E1 osteoblasts exhibited distorted morphology on the surface of PPF and BP/PPF cement, whereas their cell pseudopodia extended, displaying stellate or spindle. In contrast, simple PPF bone cement exhibits a smooth surface and fewer aggregated cells. The micrographs reveal that bone cement cells are closely packed together after the addition of BPNSs. Thus, it can be inferred that BPNSs promote cell adhesion to the material surface. Moreover, the inclusion of BPNSs shows a significant boost in the osteogenic activity of MC3T3-E1 cells compared to PPF cement. This may be due to the oxidation of BPNSs to phosphate ions to promote the proliferation and osteogenic differentiation of bone marrow mesenchymal stem cells [56]. Similar osteogenic effects were obtained when BPNSs were added to other materials [20]. We did not test the effect of black phosphorus on the osteogenic and cell viability of MC3T3-E1 cells under 808 nm laser irradiation because direct radiation exposure may damage the cells. However, osteoblasts display a heat shock response under the photothermal impact, causing upregulation of heat shock proteins to enhance osteogenic differentiation [57, 58].

This study had several limitations and must be viewed in that light. Black phosphorus exhibits high toxicity at elevated concentrations, yet its degradation into phosphate is innocuous. The bone cement formulated in this investigation indicated prompt elution during the initial two weeks. Nonetheless, the outcomes of cytotoxicity assessments demonstrated that the cell survival ratio remained within acceptable boundaries. Degradation behaviours of BP/PPF, including porosity, PH, and mechanical properties, are worthy of attention, which will be further discussed in a subsequent study. Further experimentation will be conducted with optimization of bone cement prescription. Our team aims to achieve anti-infection while completing bone cell crawling by using 3D printing technology to create BP/PPF scaffolds that are loaded with antibiotics. This will enable functional integration of the materials in subsequent stages.

## Conclusion

A novel PPF bone cement model was created using different concentrations of black phosphorus and used in bone tissue engineering. BP/PPF exhibited mechanical properties similar to that of bone: outstanding photothermal properties, cytocompatibility, and osteoinductivity. BP/PPF serves as an effective degradable bone cement and holds great potential in the field of bone regeneration.

### Supplementary Information

**Additional file 1**. **Figure S1**: ^1^HNMR spectrum of PPF.

**Additional file 2**. **Figure S2**: Twice gel permeation chromatography of PPF.

**Additional file 3**. **Table S1**: Average molecular mass of PPF.

## Data Availability

The datasets analysed during the current study are available from the corresponding author on reasonable request.

## Abbreviations

PPF: Poly(propylene fumarate)
PMMA: Polymethylmethacrylate
2D: Two-dimensional
BPN: Black phosphorus nanomaterial
CSD: Critical size defect
NMP: *N*-Methylpyrrolidone
BPO: O-Benzoyl peroxide
DMT: 1,4-Benzenedicarboxylate
PTFE: Polytetrafluoroethylene
