# Supplementary figures and images for: Incorporation of black phosphorus nanosheets into poly(propylene fumarate) biodegradable bone cement to enhance bioactivity and osteogenesis
Source: J Orthop Surg Res. 2024 Jan 30;19:98. doi: 10.1186/s13018-024-04566-6 (PMC10829309; doi:10.1186/s13018-024-04566-6)

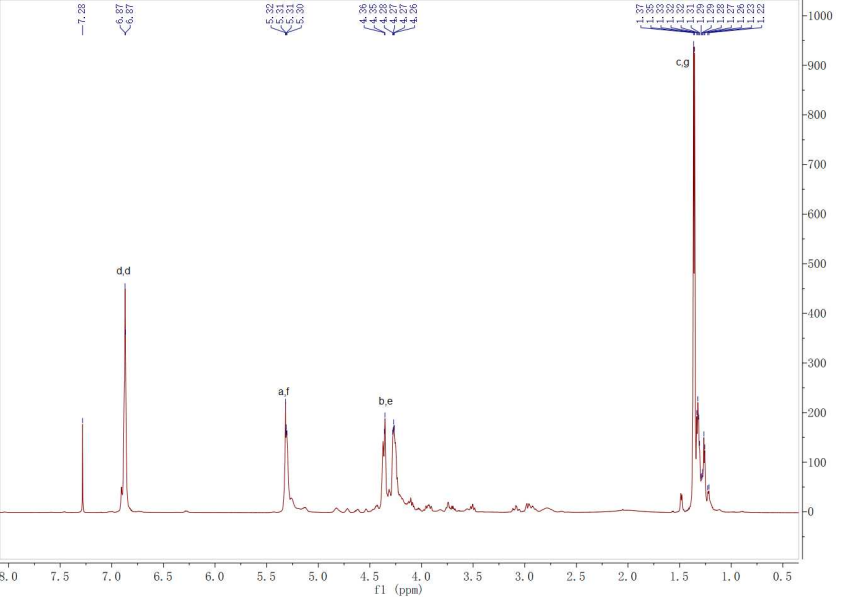

Supplement: Supplementary file 1 — Additional file 1. Figure S1: 1HNMR spectrum of PPF. [file 13018_2024_4566_MOESM1_ESM.png]

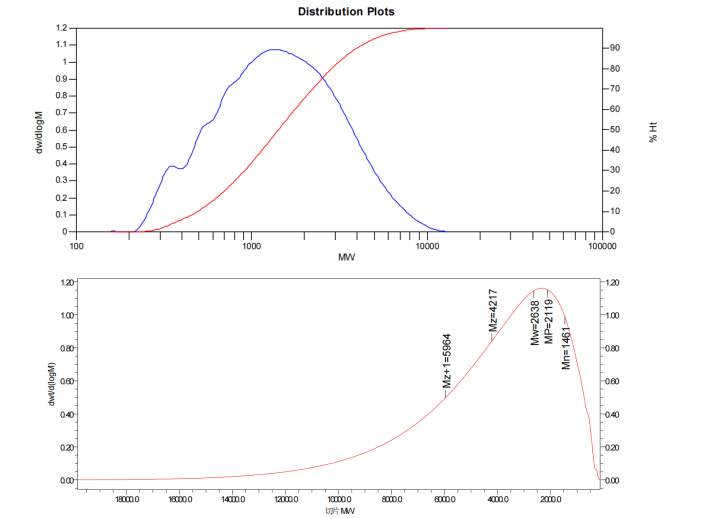

Supplement: Supplementary file 2 — Additional file 2. Figure S2: Twice gel permeation chromatography of PPF. [file 13018_2024_4566_MOESM2_ESM.png]
