# Supplementary material for: Incorporation of black phosphorus nanosheets into poly(propylene fumarate) biodegradable bone cement to enhance bioactivity and osteogenesis
Source: J Orthop Surg Res. 2024 Jan 30;19:98. doi: 10.1186/s13018-024-04566-6 (PMC10829309; doi:10.1186/s13018-024-04566-6)
Supplement: Supplementary file 3 — Additional file 3. Table S1: Average molecular mass of PPF. [file 13018_2024_4566_MOESM3_ESM.docx]

| Group | Mn | Mw | PD |
| --- | --- | --- | --- |
| PPF1 | 1416 | 2638 | 1.805 |
| PPF2 | 1675 | 2500 | 1.493 |
| PPF3 | 1041 | 1881 | 1.807 |
| mean±SD | 1392±323 | 2340±403 | 1.70±0.18 |

Table S1. Average molecular mass of PPF
